# Novel miRNA biomarkers for alveolar echinococcosis: sequencing and clinical validation

**DOI:** 10.1017/S0031182024001367

**Published:** 2024-11

**Authors:** Jideng Ma, Zian Li, Lanmin Liu, Xiaoqin Luo, Xiaoya Ma, Yumei Zhang, Lei Jiang, Xiangren A

**Affiliations:** 1 Qinghai University, Xining 810001, China; 2 Qinghai Provincial Peoples Hospital, Xining 810007, China; 3 Northwest Plateau Institute of Biology, Chinese Academy of Sciences, Xining 810000, China

**Keywords:** alveolar echinococcosis, diagnostic biomarker, microRNA, miRNA sequencing, novel predicted microRNA

## Abstract

This study aimed to explore extracellular microRNA derived from *Echinococcus multilocularis* (EM) in the plasma of patients with alveolar echinococcosis (AE) and assess its potential as a diagnostic biomarker. EM-derived miRNAs were identified in plasma samples from 20 AE patients through miRNA sequencing. Three novel miRNA molecules (emu-miR-novel 1, 2 and 3) were predicted through bioinformatic analysis to elucidate their chromosomal locations, secondary structures and precursor forms. Subsequently, plasma samples from 30 AE patients and 30 controls were utilized to establish an assay *via* stem-loop reverse transcription PCR, optimizing primers, reaction systems, and conditions to assess cross-reactivity and sensitivity. Clinical validation revealed that emu-miR-novel 1 had the highest diagnostic accuracy, with an area under the curve (AUC) of 0.8994, a *P* value of less than 0.0001, a sensitivity of 83.3%, and a specificity of 86.7%. Statistically significant differences were observed between the groups for emu-miR-novel 1 (*P* < 0.05), whereas emu-miR-novel 2 and 3 showed AUC values of 0.7922 and 0.6883, with *P* values of 0.0001 and 0.012, respectively, indicating no significant difference between groups (*P* > 0.05). Furthermore, the assay showed no cross-reactivity with samples from 18 common viruses, 4 parasitic infections, and miRNAs from AE sequenced from 8 species, confirming its high specificity. Emu-miR-novel 1 exhibited a sensitivity of 1 femtomolar. Emu-miR-novel 1 holds promise as a key diagnostic tool for AE, offering a novel perspective and approach for disease diagnosis.

## Graphical abstract

**Figure:**
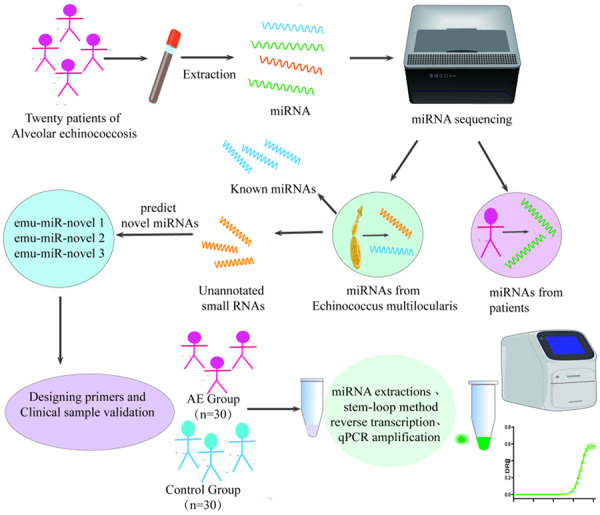

## Introduction

Alveolar echinococcosis (AE) is a parasitic disease caused by the larva of the tapeworm *Echinococcus multilocularis* (EM), which primarily infects the liver (Craig *et al*., 2017). However, it can gradually infiltrate nearby tissues and organs, and even spread to other parts of the body such as the brain and lungs, leading to serious complications (Eckert and Deplazes, 2004). The disease is mainly found in certain areas of the Northern Hemisphere, including Central Europe, Alaska, Canada, Siberia, and some states in the United States. In China, AE is mainly prevalent in western regions such as Tibet and Qinghai, where the average infection rate is 1.08% and the morbidity rate is 0.5 to 5.0%. In some areas of the Tibet Plateau, the morbidity rate can reach 5.0 to 10.0% (Gessese, 2020). About 66 million people are at risk in these areas, causing direct economic losses of 3 billion yuan annually (Giraudoux *et al*., 2019). Due to its high pathogenicity, disability rate and mortality rate, untreated AE patients have a 10-year mortality rate of up to 94%, and it is therefore also known as ‘parasitic cancer’ (Brunetti *et al*., 2010).

AE has become a significant global health concern, posing a serious threat to global public health and economic development. The World Health Organization (WHO) has listed echinococcosis as one of the 17 diseases to be controlled or eliminated by 2050 (Deplazes *et al*., 2017). Echinococcosis has unique biological characteristics and requires various surgical methods. Improper diagnosis, surgery, and drug treatment can lead to multiple complications and high recurrence rates. In some hospitals, complication and recurrence rates can even exceed 30% (W *et al*., 2003). If liver hydatid disease patients cannot receive effective diagnosis and treatment at their first visit, the risk of multiple surgeries will greatly increase (Bulakci *et al*., 2016). The prognosis and medical expenses of patients depend largely on the early detection of parasitic infection, individual immune status, and the level of medical services. Therefore, it is crucial to use simple and accurate methods for early diagnosis of AE.

Currently, the diagnosis of echinococcosis mainly relies on ultrasound examination, computed tomography (CT), magnetic resonance imaging (MRI), and 18F-fluorodeoxyglucose positron emission tomography (PET) (Bulakci *et al*., 2016). Immunological methods have some value in the diagnosis of echinococcosis, but their clinical utility is limited due to varying levels and stability of different immunological types, antigens and antibodies at different stages of infection, especially in early or mild cases (Díaz, 2017). Molecular methods have high sensitivity and specificity, and can provide timely and accurate diagnosis of AE, especially when other diagnostic methods are uncertain (Z *et al*., 2020). Numerous studies have shown that microRNAs play an important role in the interaction between the host and the parasite during Echinococcus infections” (Cai *et al*., 2016; Macchiaroli *et al*., 2021). Therefore, microRNAs have been proposed as a new diagnostic biomarker for echinococcosis. In addition, there are some new diagnostic technologies under research, such as nanoparticle technology, optical sensor technology and electrochemical sensor technology, which have the characteristics of rapid, convenient, sensitive and high specificity, and are expected to become vital diagnostic methods for echinococcosis in the future (Deplazes *et al*., 2017; Siles-Lucas *et al*., 2017*a*).

MicroRNAs (miRNAs) are non-coding RNA molecules with a length of about 20–22 nucleotides, which are associated with various diseases (Sun *et al*., 2022). The miRNA expression profile can serve as a biomarker for various tumours, liver injury and other diseases, and has the potential for early diagnosis, classification and prognosis (Enright *et al*., 2003; Toor *et al*., 2022; Moosa *et al*., 2023). Some studies have shown that miRNAs derived from Echinococcus are present in the serum and plasma of infected hosts (Siles-Lucas *et al*., 2017*b*; Paul *et al*., 2020; Wang *et al*., 2024). After AE infection, two types of miRNA exist in the blood, one derived from the host and the other from Echinococcus. The role of host miRNA is to regulate the host's cellular processes, which may indirectly affect the parasites; however, Echinococcus miRNA appears to facilitate the growth and survival of the parasites within the host. It has been suggested that EM-derived miRNA should be prioritized as a biomarker over host-derived miRNA due to differences in abundance and susceptibility to interference (Alizadeh *et al*., 2020; He *et al*., 2020; Paul *et al*., 2020). However, the current research on miRNA mainly focuses on screening differential miRNAs between disease and control groups, and compared with host-derived miRNA, the content of EM-derived miRNA is very low, making it difficult to screen using traditional methods (Cucher *et al*., 2011, 2015; Grecco *et al*., 2023). In addition, there are fewer studies on miRNAs as subjects in patients with AE, with most studies focusing on differences in miRNAs in *Echinococcus granulosus vs* animal serum (Macchiaroli *et al*., 2015; Guo and Zheng, 2017; Pérez *et al*., 2019). Although studies have explored the potential of EM-derived miRNAs as diagnostic biomarkers, they have mostly been limited to animal experiments (Alizadeh *et al*., 2020; Xiao *et al*., 2022; Cucher *et al*., 2023; Karami *et al*., 2023; Wang *et al*., 2024). However, in this study, EM-derived miRNAs were screened from human plasma for the first time using miRNA sequencing technology and these were newly predicted miRNAs, which have not been reported elsewhere. It was also validated by qPCR in clinical samples. Our findings provide new perspectives and methods for the diagnosis of AE and further enrich the research in this field.

## Materials and methods

### Patients and ethics

From January 2023 to December 2023, 20 AE patients' plasma samples were collected from the Qinghai Provincial People's Hospital for microRNA sequencing. Between March 2024 and August 2024, 30 AE patients and non-AE patients admitted during the same period were collected as clinical validation samples in Qinghai Provincial People's Hospital. All AE patients were diagnosed based on preoperative radiological examinations and intraoperative and postoperative pathological examinations. A total of 10 mL of whole blood samples treated with EDTA-K2 were collected from both patients and the healthy control group, centrifuged at 3000 rpm for 10 minutes, and stored at −80°C. Clinical information, including age, sex, lesion diameter, Echinococcus antibody, biochemical indicators and imaging data, was collected for all samples. The study was approved by the Research and Ethics Committee of Qinghai Provincial People's Hospital (License No. 2023-069) and obtained the permission for the collection of Chinese human genetic resources (License No. 2023CJ0212). All participants provided written informed consent before participating in the study

The 20 AE patients selected for miRNA sequencing all showed positive results in the radiological examination and were collected before surgery. Among these cases, there were 8 male and 12 female patients, aged 9–67 years with a mean age of 40.5 ± 13.4 years. Nineteen patients were of Tibetan ethnicity, and only one was of Han ethnicity. All selected patients had been diagnosed with intermediate to advanced hepatic hydatid disease; among whom, 10 cases showed involvement of adjacent organs and tissues, including 3 cases of the abdomen, 1 case of the colon, 1 case of the kidney, 1 case of the pancreas, 1 case of the gallbladder, 1 case of the diaphragm, 1 case of the femur and 1 case of the pelvic cavity. In addition, 10 patients had distant metastases with various sites and conditions, including 3 cases with lung and left-brain metastases, 2 cases with lung metastases, 2 cases with right lung metastases, 1 case with left lung metastases, 1 case with left brain metastases, and 1 case with right brain metastases. It is worth noting that the recurrence rate of AE is high. Among the samples in this study, 10 patients were first-time patients, and 10 patients had a recurrence after surgical treatment, with recurrence periods ranging from 2 to 17 years. In terms of lesion distribution, 15 patients had multiple lesions, while the remaining 5 had solitary lesions. Eighteen patients tested positive for hydatid antibodies, and 2 tested negative. The characteristics of all patients are shown in Table 1.

**Table 1.** Basic information of clinical samples for miRNA sequencing

Sample ID	Age (years)	Gender	Nationality	Lesion location	S/M	I/R	RT (years)	AEAb (COI)
AE1	66	Female	Tibetan	Right liver; right lung, colon	S	R	5	2.71
AE2	39	Female	Tibetan	Liver; right brain	M	R	7	3.4
AE3	67	Male	Tibetan	Right liver; Both lungs; left brain	M	R	4	5.37
AE4	27	Female	Tibetan	Right liver; Both lungs	M	I	–	0.46
AE5	33	Female	Tibetan	Liver, Both lungs; abdomen	M	I	–	4.23
AE6	59	Female	Tibetan	Liver; abdomen	M	R	5	1.35
AE7	34	Male	Tibetan	Both lungs; right liver; left brain; right kidney;	M	R	4	2.53
AE8	33	Female	Tibetan	Liver; Both lungs; left brain	M	R	5	5.69
AE9	43	Male	Han	Liver; left brain; pancreas	M	R	17	2.45
AE10	35	Male	Tibetan	Right liver S3; left lung	M	I	–	3.16
AE11	42	Female	Tibetan	Right liver; left liver	M	I	–	4.75
AE12	37	Female	Tibetan	Right liver	S	I	–	2.32
AE13	52	Female	Tibetan	Right liver; Both lung; gallbladder	M	I	–	7.42
AE14	45	Male	Tibetan	Right liver	M	R	6	6.63
AE15	9	Male	Tibetan	Liver S5; diaphragm	S	I	–	10.07
AE16	40	Male	Tibetan	Both liver; right lung	M	I	–	0.96
AE17	43	Female	Tibetan	Right liver; pelvis; bilateral femurs	M	R	2	4.11
AE18	29	Female	Tibetan	Left liver; pelvis	M	R	4	1.85
AE19	41	Male	Tibetan	Liver S7	S	I	–	1.45
AE20	35	Female	Tibetan	Liver S5; abdomen	S	I	–	6.28

***Notes*: S**, Single lesion; **M**, Multiple lesions; **I**, Initial infection; **R**, Recurrence; **RT**, Time to recurrence after surgery; **AEAb**, Echinococcus antibody.

### MicroRNA sequencing

Total RNA was extracted from the samples using TRIzol reagent (Invitrogen, CA, USA) according to the manufacturer's protocol. The concentration of total RNA was measured using NanoDrop 2000 (Thermo Fisher Scientific, USA), and the quality of RNA was evaluated using Agilent 2100 Bioanalyzer (Agilent Technologies, USA). For the construction of small RNA libraries, 1 *μ*g of total RNA was used for each sample, and the NEB Next Small RNA Library Prep Set for Illumina kit (Catalog No. NEB#E7330S, NEB, USA) was used according to the manufacturer's recommendations. In brief, sequencing adapters were ligated to both ends of the RNA, followed by reverse transcription of adapter-ligated RNA into cDNA and PCR amplification. Subsequently, gel electrophoresis was performed, and the small RNA library was purified from PCR products ranging from 140 to 160 bp. The quality of the library was assessed using the Agilent Bioanalyzer 2100 system. Sequencing was performed on the Illumina NovaSeq 6000 platform, generating 150 bp paired-end reads. Small RNA sequencing and subsequent analysis were performed by OE Biotech Co., Ltd. (Shanghai, China).

### Sequencing data analysis

(1) Base Calling and Initial Filtering**:** Raw sequencing reads were processed using the base calling algorithm implemented in the Illumina software suite. We employed FastQC version 0.11.5 for initial quality assessment of the reads. Low-quality reads were filtered out using the Fastx-Toolkit version 0.0.13 (Tino, 2009) with parameters -q 20 -p 80 to ensure high-quality sequences for further analysis. Primer contaminants and poly (A) tails were removed using Cutadapt version 1.14, ensuring clean reads for subsequent steps. (2) Read Length and Adapter Filtering: The clean reads were further refined by excluding those that did not meet the expected read length criteria of 15 to 41 nucleotides, as well as those lacking the 3′ adapter and insert tag. (3) Annotation and Alignment (Altschul *et al*., 1990): The high-quality reads were aligned to the reference genome using Bowtie version 1.1.1 with parameters -f -p 20 optimized for sensitive alignment. Non-coding RNAs, including rRNAs, tRNAs, snRNAs and snoRNAs, were annotated by aligning the reads against the Rfam database version 10.1 (Griffiths-Jones *et al*., 2003) and the NCBI GenBank database. (4) miRNA Identification and Expression Analysis: Known miRNAs were identified by aligning the clean reads to the miRBase database version 21 using the BLAST + suite. The expression patterns of these known miRNAs across different samples were analysed using the DESeq2 package in R version 1.22.2, which employs a *P* value threshold of *P*adj < 0.05 and a fold change threshold of |log2FoldChange|>1 for statistical significance. (5) Prediction of Novel miRNAs: Unannotated small RNAs were analysed using mirDeep2 (Friedlander *et al*., 2012) to predict novel miRNAs (Akhtar *et al*., 2019). The software was run with parameters set to identify pre-miRNA hairpin structures and miRNA star sequences based on the miRBase database version 21(Griffiths-Jones *et al*., 2008). (6) Differential Expression Analysis: Differentially expressed miRNAs were identified with a *P* value threshold of < 0.05. For experiments with biological replicates, the DESeq2 package was used, while for experiments without replicates, the DEGseq package version 1.36 (Sun *et al*., 2013). One was employed with parameters *P*adj<0.05, |log2FoldChange|>1 to calculate the *P* value. The detailed process of sequencing data analysis is summarized in a flow chart (Fig. 2C)

Then, the reads were aligned to the human genome, and the unaligned sequences were subsequently aligned to the EM genome. Due to the limited alignment results with EM, the bowtie mismatch was set as 0 when aligning to the human genome to obtain more sequences that were not aligned to humans. Additionally, analysis was performed on the Rfam database, reference transcripts, repeat sequences and miRBase database, to predict novel miRNAs, the bowtie mismatch was set as 2 to improve accuracy (Friedlander *et al*., 2012). As EM contains 68 mature bodies, new miRNA prediction was also performed. Based on the results of previously known miRNAs and newly predicted miRNAs, quantitative analysis was performed (Tino, 2009). miRDeep2 was used to analyse unannotated reads, and the corresponding miRNA* sequences and miRNA mature sequences were determined based on the pre-miRNA hairpin structure and the miRBase database, thereby predicting new miRNAs (Akhtar *et al*., 2019). EM genomes using sequence information from NCBI as linked below: (1) genome = https://ftp.ncbi.nlm.nih.gov/genomes/all/GCA/000/469/725/GCA_000469725.3_EMULTI002/GCA_000469725.3_EMULTI002_genomic.fna.gz; (2) genome.gff = https://ftp.ncbi.nlm.nih.gov/genomes/all/GCA/000/469/725/GCA_000469725.3_EMULTI002/GCA_000469725.3_EMULTI002_genomic.gff.gz

### Primer design and reverse transcription reactions

MiRNA from patient plasma samples was extracted using the BIOG Plasma Serum miRNA Extraction Kit (Biodao, 51076, Changzhou). Post-extraction, the RNA concentration and purity were assessed using a Thermo Fisher NANODROP instrument, and the RNA was quantified to 200 ng for subsequent stem-loop reverse transcriptions. The stem-and-loop method primers were sourced from Shanghai Bioengineering Co, and details are presented in Table 2. The cDNA synthesis *via* the stem-and-loop method was conducted using the miRNA First Strand cDNA Synthesis Kit (Bioengineering Co., Ltd., #B532453, Shanghai, China). For the stem-loop reverse transcription, 1 *μ*L of a 8 *μ*m RT primer was added, following the remaining steps as outlined in the protocol.

**Table 2.** Relevant Information and stem-loop primers of Novel miRNAs

miRNA_id	emu-miR-novel1	emu-miR-novel2	emu-miR-novel3	U6
calibrators	ATGAGAATGTGGGGGATAAGC	ACTACTGGGAGTCTCGAGGAGG	ATCTATGGCTGGCAGAGCAC	GCTTCGGCAGCACATATACTAAAATTGG
F primer sequence	TCACGAGCATGAGAATGTGGGG	CTCGACCGACTACTGGGAGTCT	TGGTTCGCATCTATGGCTGGC	CTCGCTTCGGCAGCACA
R primer sequence	ATCCAGTGCAGGGTCCGAGG	ATCCAGTGCAGGGTCCGAGG	ATCCAGTGCAGGGTCCGAGG	AACGCTTCACGAATTTGCGT
RT Primer sequence	GTCGTATCCAGTGCAGGGTCCGAGGT ATTCGCACTGGATACGACGCTTAT	GTCGTATCCAGTGCAGGGTCCGAGGT ATTCGCACTGGATACGACCCTCCT	GTCGTATCCAGTGCAGGGTCCGAGGTATT CGCACTGGATACGACGTGCTC	CTCAACTGGTGTCGTGGAGTCGGCAAT TCAGTTGAGAAAAATATG
Chrom	LN902846.1	LN902843.1	LN902842.1	
Start	167 997	11 109 291	14 628 338	
End	168 052	11 109 341	14 628 394	
Strand	−	+	+	
Mature sequence	AUGAGAAUGUGGGGGAUAAGC	ACUACUGGGAGUCUCGAGGAGG	AUCUAUGGCUGGCAGAGCAC	
Stars sequence	UUCUCUCCCAUCCCAUCA	UCAAACGAGAAGGCCUACCAGUCA	GAUGAAGCCUGGGUGU	
Precursor sequence	AUGAGAAUGUGGGGGAUAAGCAUGAGCCUAUAUCUCCUUCUCUCCCAUCCCAUCA	ACUACUGGGAGUCUCGAGGAGGACACUCAAACGAGAAGGCCUACCAGUCA	AUCUAUGGCUGGCAGAGCACCAAACUCAACUUGAGGUGGUGAUGAAGCCUGGGUGU	

### Quantitative PCR

Quantitative PCR was performed using Yarui Biotech's Real-Time Quantitative PCR (qPCR) (MA-6000) instrument (China) and Takara Bio Inc (Japan) TB Green® Premix Ex Taq™ II FAST qPCR (RR830B). U6 was used as an endogenous control for miRNA amplification. The reaction volume was 20 *μ*L, which consisted of 5 *μ*L of qPCR mastermix, 10 *μ*L of cDNA template (diluted 10-fold), 1 *μ*L of a 4 *μ*m primer mixture (92 *μ*L of DEPC water, 4 *μ*L of forward primer and 4 *μ*L of reverse primer) and 4 *μ*L of DEPC water. Reaction conditions were as follows: pre-denaturation at 95°C for 3 min, followed by 41 cycles at 95°C for 5 s, 55°C for 5 s and 72°C for 20 s. Addition of the dissolution curves. Relative miRNA expression levels were calculated using the 2-ΔΔCt method. All samples and blanks were analysed in triplicate.

### Cross reactivity

Cross-reactivity with miRNAs of parasitic and origin of ‘Em’ miRNA: 4 samples of clinically confirmed parasitic infections were collected from Qinghai Provincial People‘s Hospital and Qinghai Provincial Women’s and Children's Hospital, including one sample each of *Plasmodium*, *pig tapeworm*, *Ascaris lumbricoides* and *Clonorchis sinensis* infections. A 500 *μ*L plasma sample was collected from each and immediately placed at −80°C. Extraction, reverse transcription, and qPCR steps were performed simultaneously with the samples from AE patients to reduce the influence of external variables on the experimental results. In addition, due to the high degree of sequence similarity between miRNA family members, this may lead to difficulties in distinguishing individual miRNAs during qPCR detection, affecting the specificity of the assay. To address this issue, the researchers performed specificity analyses with standards of 5 known miRNAs and 3 unknown miRNAs. The sequences of the 3 unknown miRNAs were as follows: emu-miR-1-3p (TGGAATGTTGTGAAGTATGT), emu-miR-10-5p (CACCCTGTAGACCCGAGTTTGA), emu-miR-7-5p (TGGAAGACTGGTGATATGTTGT), emu-miR-9-5p (TCTTTGGTTATCTAGCTGTGTG) and emu-let-7-3p (TCTTTGGTTATCTAGCTGTGTG). These standards were synthesized by Bioengineering Co. (Shanghai, China). All 'EM'-derived sequence miRNAs were diluted to 1 pm for detection. With this approach, researchers were able to evaluate and improve the specificity of the assays. This improvement allowed for more accurate identification and differentiation of miRNAs from different parasitic infections, providing more reliable molecular markers for clinical diagnosis and treatment.

Cross-reactivity with 18 common clinical viruses: to avoid interference from non-specific amplification in PCR results, cross-reaction tests were conducted to identify and evaluate potential interfering substances, ensuring the accuracy and reliability of PCR detection. In this study, liquid nucleic acid, quality control samples of 18 common clinical viruses were used as non-specific controls. MiRNA extraction, tail addition and qPCR reaction steps were performed under the same conditions as clinical sample detection to verify the specificity of emu-miR-novell-1-F and emu-miR-novell-2-F primers. To verify the effectiveness of quality control samples, detection was performed strictly according to the instructions of the corresponding reagents. The 18 viruses included Adenovirus 1 (ADV1), Norovirus (NV), Mycoplasma pneumoniae (MP), Human coronavirus HKU1 (HCoV-HKU1), Respiratory syncytial virus A (RSV-A), Respiratory syncytial virus B (RSV-B), Adenovirus 3 (ADV3), Cytomegalovirus (HCMV), Adenovirus 4 (ADV4), Enterovirus Type 71 (EV71), Adenovirus 5 (ADV5), Tuberculosis (TB), Adenovirus 11 (ADV11), Group A rotavirus (RVA), Adenovirus 7 (ADV7), COVID-19 (B.1.351), Enterovirus (EV), and Chlamydia pneumoniae (CP).

### Sensitivity analysis of emu-miR-novel 1

A series of different concentration points, including 1 nm, 100 pm, 10 pm, 1 pm, 100 fm, 10 fm, 1 fm and 100 am, was created by gradient dilution of the standards of emu-miR-novel-1. These concentration points represent different dilutions or concentration levels. Other assay conditions were consistent with those used for clinical samples. DEPC water was used as a template-free control. Three replicates were performed for all assays.

### Data analysis

All results are presented as mean ± s.e.M, ROC analysis was performed using GraphPad Prism version 10.1 (GraphPad Inc., La Jolla, CA, USA). Creating diagrams was done with Adobe Illustrator software. Statistical analyses were done by using GraphPad Prism and SPSS 26 (SPSS Inc., Chicago, IL, USA) software. Differences between groups were analysed by *t*-test. The significance level was set at 0.05.

## Results

### Discovery of diagnostic biomarkers

**Figure 1.**
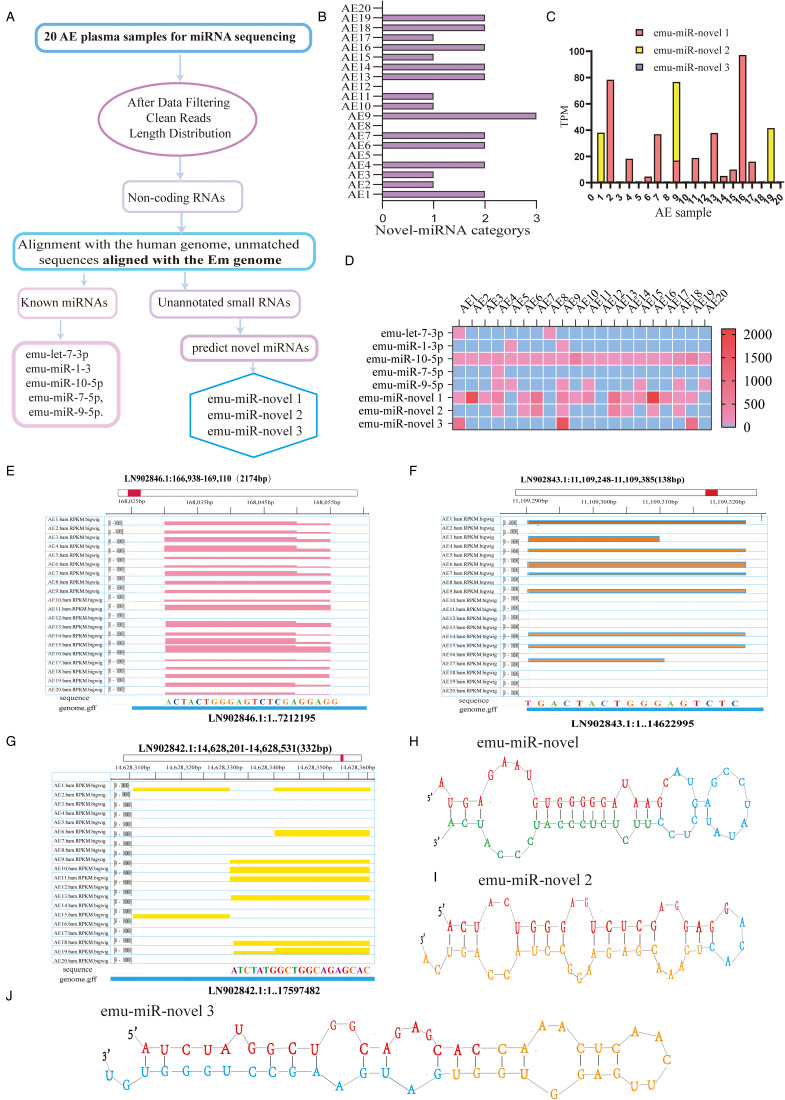
Discovery of diagnostic biomarkers. Shows the workflow of miRNA sequencing, outlining the various steps in the sequencing process. (B) Describes the novel miRNA types detected in each sample, providing the number of novel miRNAs found in each sample. (C) Shows the expression levels of the newly predicted miRNAs, quantifying their presence in the samples. (D) Heatmap of the counts of 8 miRNAs of *Echinococcus multilocularis* origin sequenced in 20 AE patients. (E–G) Chromosomal positions of emu-miR-novel 1, emu-miR-novel 2, and emu-miR-novel 3 after alignment with the reference genome, respectively. (H–J) Predicted hairpin structures (secondary structures) of emu-miR-novel 1, emu-miR-novel 2 and emu-miR-novel 3, respectively, are shown, demonstrating the precise localisation of predicted or known miRNAs in the hairpin structures.

**Figure 2.**
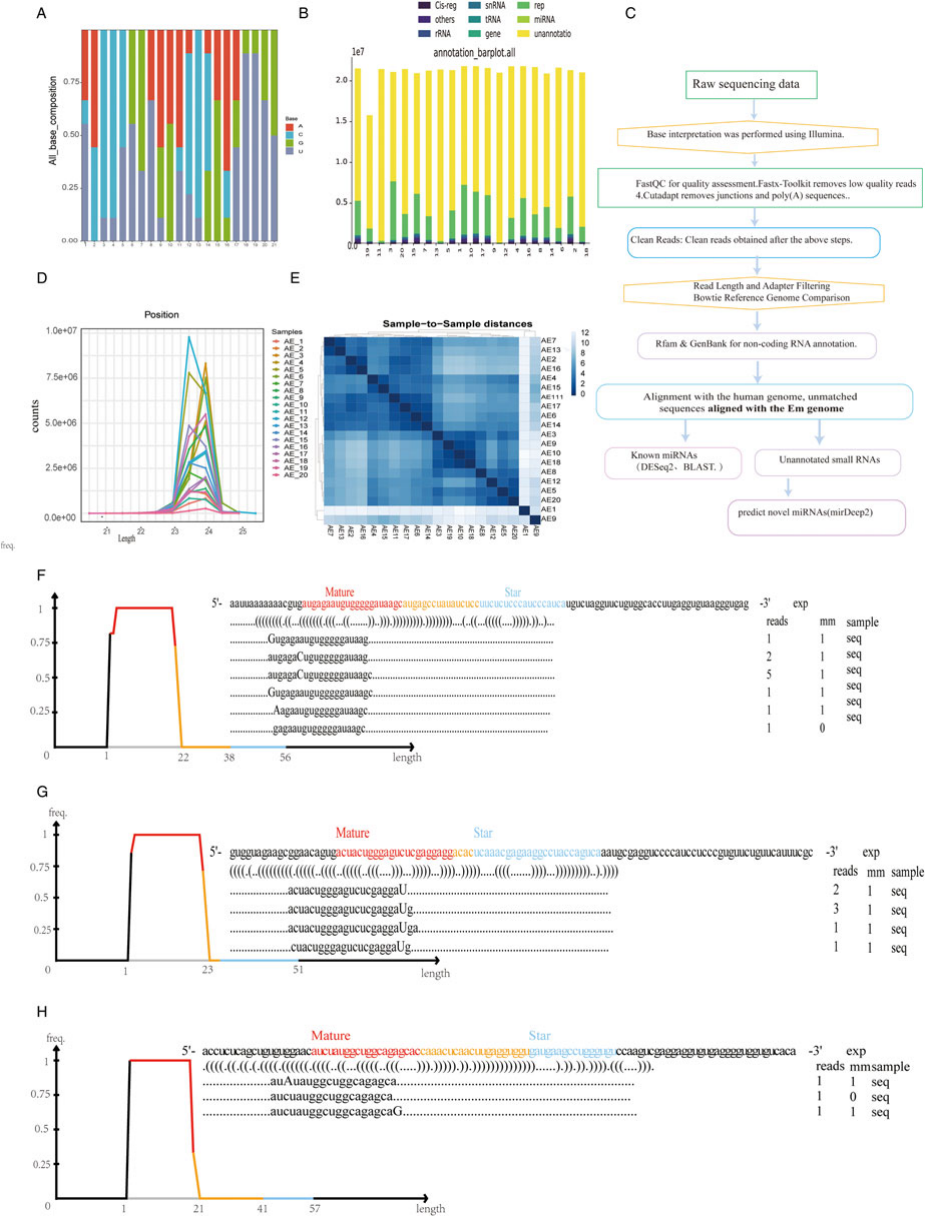
Legends: Supplementary Infographic of miRNA Sequencing Data. (A) Base Composition Plot This plot illustrates the nucleotide composition of the sequenced miRNAs, providing insight into the base distribution across the samples. (B) Taxonomic Annotation Histogram The histogram presents the taxonomic annotations of the identified miRNAs, categorized by their biological origin, highlighting the diversity of miRNA sources. (C) Sequencing Data Analysis Flow Chart This flow chart outlines the methodology and steps followed in the analysis of the sequencing results, detailing the process from raw data to final interpretation. (D) miRNA Length Distribution Line Graph The line graph depicts the distribution of miRNA lengths, indicating the prevalence of various miRNA sizes within the study's samples. (E) Sample Cluster Analysis Plot This plot displays the results of cluster analysis among the samples, revealing patterns and relationships that may suggest sample grouping based on miRNA profiles. (F) emu-miR-novel 1 Alignment and Mismatch Plot This diagram presents the alignment of emu-miR-novel 1 with the reference genome, marking the positions and nature of any observed mismatches. (G) emu-miR-novel 2 Alignment and Mismatch Plot Similarly, this diagram shows the alignment and mismatch analysis for emu-miR-novel 2, providing a detailed comparison with the reference sequence. (H) emu-miR-novel 3 Alignment and Mismatch Plot The final diagram in the series illustrates the alignment and mismatch details for emu-miR-novel 3, completing the genomic comparison for the novel miRNAs under investigation.

Figure 1A illustrates the process of discovering novel parasitic miRNA biomarkers for diagnosis. Total RNA was extracted from the serum of 20 positive patients, and microRNA sequencing was performed. Human-derived microRNAs were filtered out, and microRNAs belonging to EM were selected. After removing known EM microRNAs, three newly discovered EM microRNAs (emu-miR-novel 1, emu-miR-novel 2, emu-miR-novel 3) were identified. The clean data volume of the 20 samples ranged from 15.8 to 21.8 million, with a genomic alignment rate ranging from 0.69% to 25.23%. Figure 2A shows a base composition graph, indicating that the base composition of the sequencing results varied greatly among different clinical samples, and most base distributions were uneven. Figure 2B is a classification annotation histogram, showing that most RNAs in the clinical samples belonged to microRNAs. Figure 2D is a line graph of miRNA length distribution, which indicates that miRNA lengths are concentrated at 21–25 nucleotides, suggesting the reliability of the screened miRNAs. Figure 2E is a clustering analysis diagram among samples, revealing that samples AE1 and AE9 have lower similarity to the other samples.

The miRNA profiling heatmap depicted in Fig. 1D reveals the quantitative assessment of 8 EM-derived miRNAs identified through sequencing analysis in a cohort of 20 patients afflicted with AE. Among these, 5 are established miRNAs: emu-let-7-3p, emu-miR-1-3p, emu-miR-10-5p, emu-miR-7-5p, and emu-miR-9-5p. Positivity for these miRNAs was defined by a count value exceeding 1. The calculated positivity rates for these known miRNAs are as follows: 10% for emu-let-7-3p, 10% for emu-miR-1-3p, 100% for emu-miR-10-5p, 5% for emu-miR-7-5p, and 35% for emu-miR-9-5p. Additionally, 3 newly predicted miRNAs, designated as emu-miR-novel 1, emu-miR-novel 2 and emu-miR-novel 3, demonstrated positivity rates of 80%, 40% and 15%, respectively. Notably, two of the samples in emu-miR-novel 1 had counts over 2000 and 1 of emu-miR-novel 3 was close to 2000. The combined positivity rate and count value of emu-miR-novel 1 is a relatively promising new diagnostic marker.

The focus of this study is on utilizing 3 newly predicted miRNAs found in the plasma of AE patients (i.e. emu-miR-novel 1, emu-miR-novel 2 and emu-miR-novel 3) as diagnostic biomarkers. Table 2 provides essential information on the 3 newly predicted RNAs, including their sequence (5′-3′), mature sequence, star sequence, precursor sequence, and coordinate information. Figure 1B illustrates the distribution of the newly discovered miRNAs across different clinical samples, with none detected in 4 clinical samples, and at least 1 detected in the remaining 16. Figure 1C displays the relative expression levels of the newly discovered miRNAs in 20 clinical samples, with emu-miR-novel 1 found in 16 positive cases, emu-miR-novel 2 found in 8 cases, and emu-miR-novel 3 found in 3 cases. emu-miR-novel 1 had the highest relative content, followed by emu-miR-novel 2, with emu-miR-novel 3 having the lowest content. Figure 1E–G depict the chromosomal locations of emu-miR-novel 1, emu-miR-novel 2, and emu-miR-novel 3 aligned with the reference genome, respectively. emu-miR-novel 1 is located at 168027-168055 bp in the EM genome, emu-miR-novel 2 at 11109290–11109325 bp, and emu-miR-novel 3 at 14628310–14628360 bp. Figure 1H–J present the predicted hairpin structures (secondary structures) for emu-miR-novel 1, emu-miR-novel 2, and emu-miR-novel 3, respectively.

By referring to Table 2, 1 can observe the sequences of the mature sequence, star sequence, and precursor sequence within the hairpin structures. Figure 2F–H depicts the alignment positions and mismatch scenarios for emu-miR-novel 1, emu-miR-novel 2, and emu-miR-novel 3 miRNAs relative to the reference genome, respectively. These figures describe the alignment of reads for each known miRNA precursor with its mature form, including mismatch statistics. The ‘Reads’ column indicates the number of reads aligned at that position, and the ‘mm’ column indicates the number of mismatched bases (mismatched bases are represented by uppercase letters in the alignment diagram).

The maturation process of miRNAs involves cleavage from a longer precursor into a mature form of 20–25 nucleotides in length, which is difficult to detect by conventional PCR methods due to its short length (Table 3). To overcome this challenge, the stem-loop method was used in the study to detect miRNAs. Supplementary Fig. 1 illustrates the principles of miRNA maturation and stem-loop reactions.

**Table 3.** Clinical sample information for PCR experiments

Category	Group	Frequency	Percent (%)
Gender	Male	14	46.67%
	Female	16	53.33%
Age groups (years)	10	2	6.67%
	11–30	4	13.33%
	31–40	11	36.67%
	41–50	7	23.3%
	51–60	3	10%
	⩾60	3	10%
Nationality	Chinese Tibetan	28	93.33%
	Chinese Han	2	6.67%
Cyst number	Singer	8	26.67%
	Multiple	22	73.33%
Involved lobe (liver)	Right	8	66.67%
	Left	3	25%
	Right and left	1	8.33%
Infected Organ	Liver	12	40%
	Multiple organs	18	60%
distant metastasis	No	17	56.67%
	Yes	13	43.3343%
	Lung	10	76.92%
	Brain	3	23.07%

### Validation of clinical samples

In the stem-loop qPCR validation cohort of 30 AE patients, the gender distribution was 14 males (46.67%) and 16 females (53.33%). The age distribution included 2 patients (6.67%) under 10 years old, 4 (13.33%) aged 11 to 30, and 3 (10%) aged over 60 years old. The ethnic composition comprised 28 Chinese Tibetans (93.33%) and 2 Han Chinese (6.67%). In terms of cyst count, 8 patients (26.67%) presented with single cysts, while 22 (73.33%) had multiple cysts. Among the types of infected organs, liver infection was identified in 12 patients (40%), while multi-organ infection was observed in 18 (60%). The distribution of affected lung lobes included 8 patients (66.67%) with involvement of the right lung, 3(25%) with the left lung, and 1 (8.33%) with bilateral involvement. Regarding distant metastases, 17 patients (56.67%) exhibited no evidence of distant metastases, while 13 (43.33%) had metastases, with 10 showing lung involvement, 3 showing brain involvemen.30 control samples were randomly selected from non-AE patients who were also being treated in the hospital and whose ages ranged from 9 to 67 years with a mean age of (39.00 ± 14.42) years. In the stem-loop qPCR validation cohort of 30 AE patients, the gender distribution was 14 males (46.67%) and 16 females (53.33%). The age distribution included 2 patients (6.67%) under 10 years old, 4 (13.33%) aged 11 to 30, and 3 (10%) aged over 60 years old. The ethnic composition comprised 28 Chinese Tibetans (93.33%) and 2 Han Chinese (6.67%). In terms of cyst count, 8 patients (26.67%) presented with single cysts, while 22(73.33%) had multiple cysts. The distribution of affected lung lobes included 8 patients (66.7%) with involvement of the right lung, 3 (25%) with the left lung, and 1 (8.33%) with bilateral involvement. Among the types of infected organs, liver infection was identified in 12 patients (40%), while multi-organ infection was observed in 18 (60%). Regarding distant metastases, 17 patients (56.67%) exhibited no evidence of distant metastases, while 13 (43.33%) had metastases, with 10 showing lung involvement, 3 showing brain involvement. Thirty control samples were randomly selected from non-AE patients who were also being treated in the hospital and whose ages ranged from 9to 67 years with a mean age of (39.00 ± 14.42) years.

**Figure 3.**
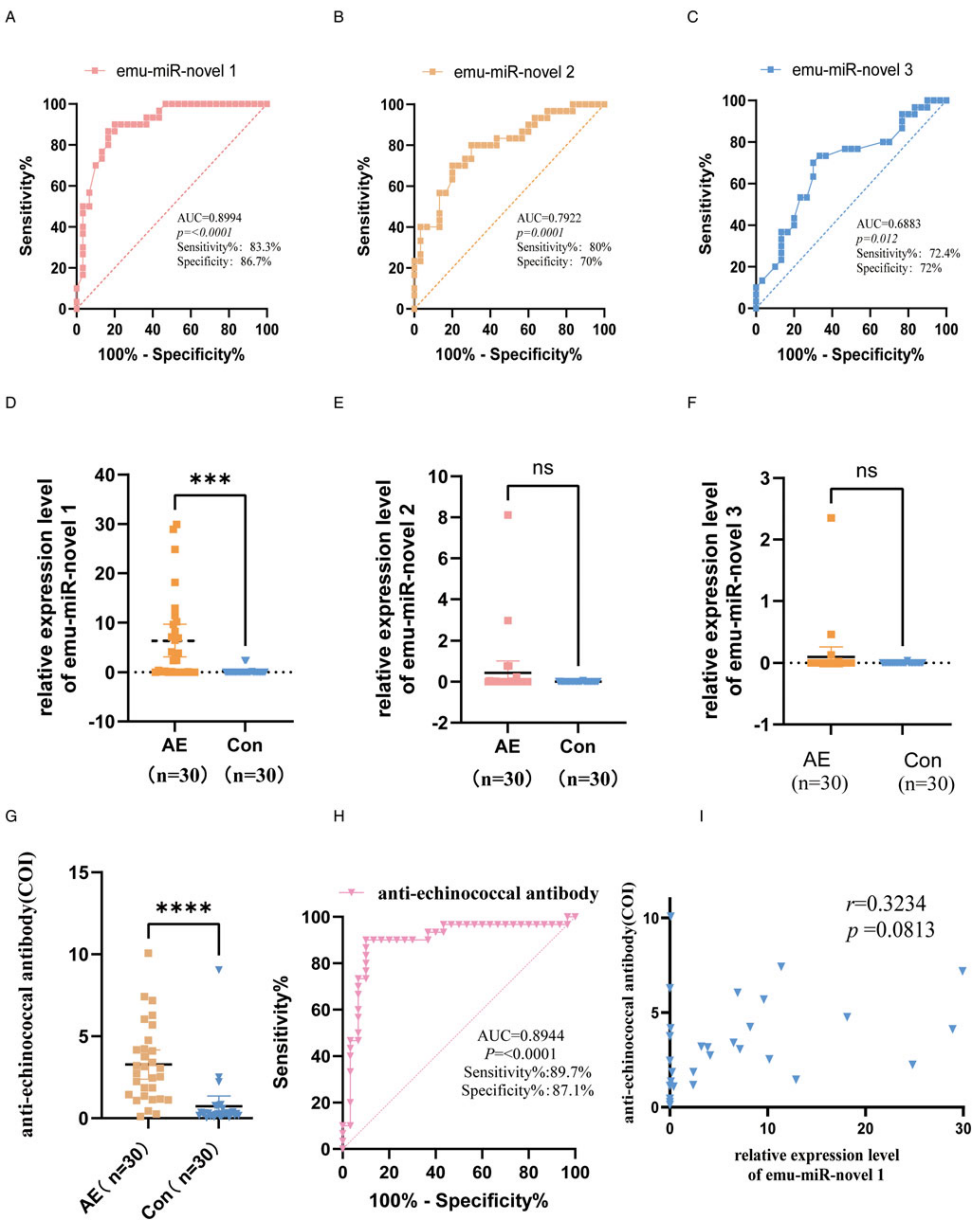
Validation of the AE diagnostic efficacy of three newly predicted miRNAs using clinical samples. (C) Representative receiver operating characteristic (ROC) curves for the detection of 3 novel microRNAs, emu-miR-novel 1, emu-miR-novel 2, and emu-miR-novel 3, derived from clinical samples. These curves illustrate the diagnostic accuracy of each microRNA as a potential biomarker for AE. (D–F) Bar charts displaying the comparative analysis of relative expression levels of emu-miR-novel 1, emu-miR-novel 2, and emu-miR-novel 3, identified through stem-loop reverse transcription polymerase chain reaction (RT-PCR), between the AE group and the control group. Statistical significance is indicated to assess the differential expression of these microRNAs in the context of AE. (G) A bar chart comparing the levels of echinococcal antibodies between the 2 cohorts of samples, highlighting the presence of antibodies as an indicator of infection in AE patients. (H) The ROC curve for the diagnosis of AE based on the presence of Echinococcus antibody, demonstrating the test's sensitivity and specificity in diagnosing AE. (I) A correlation analysis curve depicting the relationship between the relative expression levels of emu-miR-novel 1 and the levels of Echinococcus antibody, providing insights into the potential linkage between these 2 biomarkers in AE diagnosis.

In order to accurately assess the diagnostic efficacy of emu-miR-novel 1, emu-miR-novel 2 and emu-miR-novel 3 as AE biomarkers, data from clinical samples were analysed after continuous optimization of the reaction system and procedures. The results of the study are as follows: Figure 3A–C demonstrates the ROC curves of these miRNAs, which reveal the diagnostic ability of each miRNA as a potential biomarker. Among them, emu-miR-novel 1 demonstrated the highest diagnostic accuracy with an area under the ROC curve (AUC) of 0.8994, a *P* value of less than 0.0001, a sensitivity of 83.3% and a specificity of 86.7%. In contrast, emu-miR-novel 2 and emu-miR-novel 3 had AUC values of 0.7922 and 0.6883, with *P* values of 0.0001 and 0.012, respectively, showing their potential in AE diagnosis, albeit with a slightly lower accuracy than that of emu-miR-novel 1. Figure 3D–F show, by means of a bar graph, the comparative analysis of the relative expression levels of these miRNAs. The results showed that the differences between emu-miR-novel 2 and emu-miR-novel 3 were not statistically significant. In contrast, emu-miR-novel 1 showed significant up-regulated expression in AE patients. To better evaluate the diagnostic efficacy of emu-miR-novel 1, Echinococcus antibody in the 2 groups of samples was analysed, Fig. 3G showed that the difference between the 2 groups was statistically significant, and Fig. 3H showed the ROC curve for Echinococcus antibody, with an area under the AUC curve of 0.8944, a sensitivity of 89.7%, and a specificity of 87.1%. Moreover, the correlation analysis in Fig. 3I showed that the correlation coefficient between the relative expression level of emu-miR-novel 1 and the level of anti-*Echinococcus granulosus* was 0.3234, indicating a weak positive correlation.

**Figure 4.**
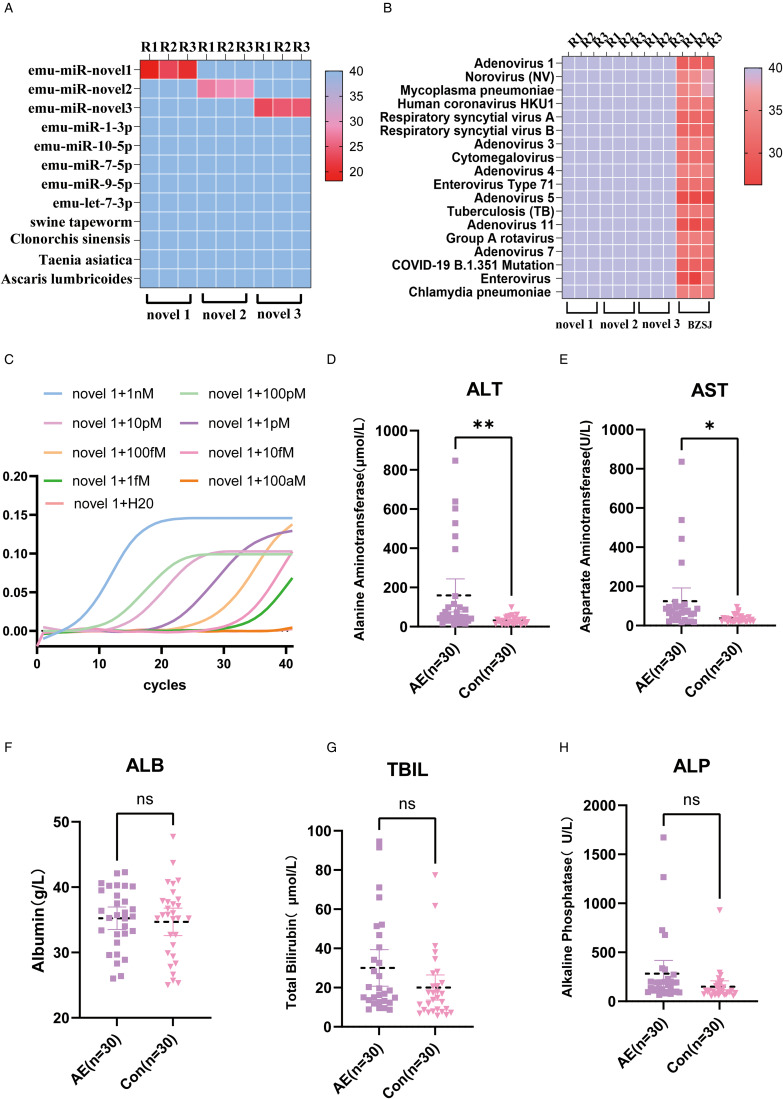
Methodology for validation of 3 new predicted miRNAs using clinical samples and laboratory metrics for validation samples. A heatmap depicting the specificity assessment of 3 primers, emu-miR-novel 1, emu-miR-novel 2, and emu-miR-novel 3, utilizing 4 distinct clinical samples of parasitic infections. These samples were tested against a panel of standards comprising 5 miRNAs derived from AE and 3 newly predicted miRNAs. The heatmap illustrates the cycle threshold (Ct) values, with R1, R2 and R3 indicating the replicate measurements for each condition. (B) A heatmap representing the specificity analysis for the same set of 3 primers when tested against standards for 18 clinically prevalent viruses. The replicate data are denoted by R1, R2, and R3 and BZSJ refers to the baseline Ct value provided by the PCR kit for the corresponding viral targets. (C) The amplification curve for emu-miR-novel 1, demonstrating the sensitivity of the assay. This curve is indicative of the method's ability to detect low levels of miRNA expression. (D–H) Histograms presenting the liver function indices – namely, alanine aminotransferase (ALT), aspartate aminotransferase (AST), albumin (ALB), total bilirubin (TBIL) and alkaline phosphatase (ALP) – for the clinical samples employed in the validation process. These indices are crucial for assessing liver health and the potential effects of parasitic infections.

Figures 4C and 4D show the results of alanine aminotransferase (ALT) and aspartate aminotransferase (AST) tests, which were significantly higher in the positive patient group than in the control group, and the differences were statistically significant (*P* < 0.05). Figure 4F–H show the total bilirubin level, plasma albumin, and alkaline phosphatase in the AE group compared with the control group, and the differences were not statistically significant (*P* > 0.05).

### Cross-reactivity

To assess the specificity of the assay results when performed with the emu-miR-novel 1, emu-miR-novel 2, and emu-miR-novel 3 primers, cross-reactivity tests were performed with 4 parasite clinical samples and other EM-derived miRNAs. As shown in Fig. 4A: the 1 pm emu-miR-novel 1, emu-miR-novel 2, and emu-miR-novel 3 standards showed positive results when tested with their corresponding stem-loop primers, and the mean and standard deviation of their Ct values for 3 replicates were 20.45 ± 1.96, 28.30 ± 0.69, and 24.09 ± 0.61, respectively. The Ct values of the other samples were higher than 40, which indicated that there was no cross-reactivity with the miRNAs of these 4 parasites and 7 other AE sources when using these 3 new biomarkers for the detection of *E. multilocularis*.

To assess the specificity of the test results, cross-reactivity tests were performed on 18 clinically common viruses and bacteria. As shown in Fig. 4B, significant amplification was observed when the 18 pathogens were detected using the corresponding kits. Three replicates of the test were performed for each sample, and the Ct values ranged from 26–36, indicating that the quality of the control samples was acceptable. However, no amplification profiles were observed when tested with the emu-miR-novel 1-F, emu-miR-novel 2-F, and emu-miR-novel 3-F primers, with Ct values above 40, suggesting that there was no cross-reactivity with the other 18 pathogens when using these 3 new biomarkers to detect AE. This indicates that all 3 primers have high specificity.

### Sensitivity analysis

Figure 4C shows the qPCR amplification curves of emu-miR-novel 1 at different concentrations: a more obvious *S*-shaped amplification curve with a plateau period can be observed at 100, 10, and 1 pm concentrations, and a better gradient is observed between the concentration and the amplification curve. When the standard was diluted to 100 fm, due to the lower template amount, the Ct value increased further, and it was difficult to detect the obvious amplification curve. At 1 fm there was only mild tailing but still a fluorescent signal. No fluorescent signal was detected using DEPC water as a no template control.

## Discussion

The laboratory diagnosis of AE confronts numerous challenges, such as the absence of distinct clinical indicators during the disease's incipient phase, which often results in a delayed patient presentation. The intricate interplay between the host and the parasite further complicates the identification of reliable biomarkers. Additionally, traditional imaging techniques exhibit limited sensitivity in early-stage detection and struggle to discern the activity of lesions. Immunological assays are also prone to cross-reactivity, which can impede accurate diagnosis (Zhang and McManus, 2006; Bulakci *et al*., 2016). Molecular diagnostic techniques, including miRNA and circulating free DNA (cfDNA), represent burgeoning areas of research. While cfDNA holds diagnostic potential for AE, its application is not without limitations (Wan *et al*., 2020; Zhao *et al*., 2021, 2024). These limitations include the extremely high detection sensitivity required for the low concentration of cfDNA in the blood, especially in the early stages of the disease. In addition, the diversity of cfDNA sources may introduce background noise, affecting specificity (Ji *et al*., 2020). A primary constraint is the intricate nature and substantial expense associated with harnessing high-throughput sequencing for cfDNA analysis, precluding its extensive application in settings with constrained resources (Ji *et al*., 2020). The pathological and genetic heterogeneity of AE may lead to differences in circulating DNA composition, affecting detection consistency, and the strict sample processing and storage requirements present practical operational challenges. Therefore, although cfDNA has potential diagnostic value, its value in clinical applications for AE is limited (Hadipour *et al*., 2023). miRNA, on the other hand, benefits from its short sequence and stability in body fluids, and can be effectively detected even at low expression levels. In terms of cost-effectiveness, miRNA detection is relatively low-cost and suitable for large-scale screening and routine applications (Paul *et al*., 2020; Hadipour *et al*., 2023). In terms of detection rate, miRNA has a high detection rate in AE. In summary, miRNA is a promising non-invasive early diagnostic and prognostic biomarker. Currently, there are few studies on the use of miRNA for AE diagnosis.

MiRNA is transcribed by RNA polymerase II, producing a primary miRNA (pri-miRNA) with a hairpin structure composed of hundreds of nucleotides. Specific motifs in the pri-miRNA sequence are recognized by the enzymes DGCR8 and Drosha, which cleave the hairpin stem to release the precursor miRNA (pre-miRNA) (Ha and Kim, 2014). Pre-miRNA is produced in the nucleus and transported to the cytoplasm by export protein 5. In the cytoplasm, pre-miRNA is further processed by RNA endonucleases DICER and TRBP, leading to the production of double-stranded miRNA with a single-stranded 3′ end containing 2–3 nucleotides. Helicase then separates the 2 strands of miRNA, allowing the mature strand to enter the RISC complex, while the other strand (called the star strand) is degraded (Treiber *et al*., 2019; Rani and Sengar, 2022). The miRNA RISC complex (miRISC) is then transported from the cytoplasm to the nucleus using Importin 8. Once in the nucleus, miRISC binds to the promoter of target genes, affecting their expression (Krol *et al*., 2010; Komatsu *et al*., 2023) (Fig. 3A).

Through miRNA prediction, 3 previously unreported miRNAs were identified, and their secondary structures, chromosomal locations, precursors, and mature forms were determined using bioinformatics analysis. Utilizing miRNAs derived from the parasite as diagnostic markers effectively ensures specificity and avoids interference from human-derived miRNAs. Sequencing results revealed that the expression rates of these 3 miRNAs were relatively high in 20 clinical samples used for sequencing, especially emu-miR-novel 1 and emu-miR-novel 2. It is speculated that designing qPCR reactions targeting these miRNAs as biomarkers would provide higher sensitivity and specificity, which could facilitate the application of these biomarkers in clinical diagnosis. The discrepancy between the failure to detect certain miRNAs in clinical samples during sequencing and their subsequent detection using PCR can be attributed to the inherent limitations of sequencing technology. Although sequencing provides high-throughput data, bioinformatics analysis is required to identify and quantify miRNAs. Low-abundance miRNAs may be omitted during this process, while qPCR, as a validation tool, can directly target specific miRNAs for quantification, enabling the detection of these previously unnoticed miRNAs.After continuously exploring and optimizing the conditions for extraction, reverse transcription, and amplification, The diagnostic precision of emu-miR-novel 1 was notably superior, as evidenced by a clinical validation study that reported an AUC score of 0.8994, a highly significant *P* value at less than 0.0001, alongside a sensitivity and specificity rates of 83.3 and 86.7%, respectively. Comparatively, emu-miR-novel 2 and 3 exhibited lower AUC scores of 0.7922 and 0.6883, with corresponding *P* values at 0.0001 and 0.012, respectively; however, these did not reach a statistically significant difference when the groups were compared (with a *P* value threshold of 0.05). Moreover, the assay demonstrated an absence of cross-reactivity with a broad range of pathogens, including 18 prevalent viruses, 4 types of parasitic infections – such as *Plasmodium*, *porcine tapeworm*, *Ascaris lumbricoides* and *Clonorchis sinensis* – and miRNAs extracted from AE across 8 different species, thereby affirming its exceptional specificity. In terms of sensitivity, emu-miR-novel 1 was capable of detecting as low as 1 femtomolar concentration.

During host–parasite interactions in AE, miRNAs are released into the bloodstream by both the host and the parasite, making them detectable and potentially valuable as diagnostic biomarkers (Macchiaroli *et al*., 2015; Paul *et al*., 2020; Orsten *et al*., 2021).Currently, research on AE and miRNAs primarily focuses on exploring the functional validation of miRNAs in mouse or other animal serum and extracellular vesicles, with few studies using miRNAs as diagnostic biomarkers for AE (Faridi *et al*., 2021; Kalifu *et al*., 2021; Mohammadi *et al*., 2021; Yang *et al*., 2021; Yu *et al*., 2021). An Iranian study (Karami *et al*., 2023) evaluated serum egr-miR-2a-3p as a potential diagnostic biomarker for cysticercosis, demonstrating that parasite-derived miRNAs can be detected with optimized methodology. The study included 31 patients with CE and 15 healthy controls, finding significantly higher expression levels of egr-miR-2a-3p in preoperative patients, with an AUC of 0.8176. However, a study (Yang *et al*., 2021), which found an AUC of 0.85 for cystic echinococcosis miRNA-21, was limited by a small sample size, potentially compromising the reliability of the results. Despite these limitations, qPCR for miRNA detection offers high sensitivity and specificity, enabling the detection of miRNAs present in small quantities in the blood. While current research primarily focuses on the functional validation of miRNAs in animal models, there is a need for studies using miRNAs as diagnostic biomarkers for AE in human samples. For instance, a 2021 study (Deping *et al*., 2021) identified miR-125b-5p as a promising diagnostic marker for AE, but its human origin limits its specificity. In contrast, a 2020 study (Ancarola *et al*., 2020) reported negative RT-qPCR results for echinococcus miRNA detection, suggesting that the miRNAs in question may not serve as robust biomarkers for the disease. The discrepancy in findings may be attributed to variations in assay methodologies and sample cohorts, underscoring the necessity for standardized approaches and careful biomarker selection. Encouragingly, a recent study (Wang *et al*., 2024) using the RCA-assisted CRISPR/Cas9 assay to detect let-7-5p in experimentally infected animals showed perfect diagnostic performance with an AUC of 100 and 100% sensitivity and specificity. Although this method has yet to be tested in human samples, it suggests that isothermal amplification methods may become the mainstream diagnostic approach for detecting AE patients in the future. In conclusion, while miRNAs hold promise as diagnostic biomarkers for AE, further research is needed to standardize detection methods and to identify biomarkers with high specificity and sensitivity. The development of robust diagnostic assays will be crucial for improving the early detection and management of AE.

The highlight of the current study is the novel miRNAs, compared with known miRNAs, newly predicted miRNAs as disease diagnostic markers exhibit a series of unique advantages. Firstly, they may show highly specific expression in certain diseases, while exhibiting low or no expression levels in normal tissues or other diseases, providing more precise biological markers for disease diagnosis. Secondly, as newly discovered molecules, they may reveal previously unrecognized disease-related gene regulatory pathways, providing new perspectives for understanding the molecular mechanisms of diseases and developing new therapeutic strategies. In addition, newly predicted miRNAs may be detectable in the early stages of disease development, which has potential value for achieving early diagnosis and intervention of diseases. They may also play a critical role in monitoring disease progression and evaluating treatment efficacy, facilitating the implementation of personalized medicine and precision treatment. Furthermore, these newly predicted miRNAs may be associated with specific subtypes of diseases, helping to classify diseases at the molecular level and evaluate prognosis. In summary, emu-miR-novel 1 not only have high value in the diagnosis of AE but also hold promise for future research into the functions and subtypes of miRNAs.

However, there are several limitations of emu-miR-novel 1 as a diagnostic marker for AE, firstly, if the miRNA is of polycomb echinococcal origin, the main sources are apoptosis or necrosis and exosomal release. The ability to detect it in blood and the amount detected are highly related to the stage of the disease, the site of the lesion, the volume of the infection, and the immune status of the individual. Therefore, there may be significant differences between different patients, and these can also vary with the time of collection. It is best to collect samples prior to administering treatment and during the onset of the disease. Secondly, according to repeated experiments, because the content of miRNA of parasite origin in human blood is very small, the total RNA extraction kits, such as the magnetic bead method, which are commonly used in the clinic, may not be able to extract miRNA effectively, and miRNA-specific extraction kits must be used. It is necessary to use miRNA-specific extraction kits, which have higher requirements for operation. This increases the cost of the experiment. Finally, because of the short sequence of miRNA, stem-loop structure and reverse transcription are also required before the amplification reaction. It also increases the cost of the reaction. In addition, because AE mainly occurs in remote areas, this method is difficult to operate and have PCR instrument and standard laboratory, so there is still a long way to go before to the clinical application. Therefore, the follow-up study plans to use isothermal amplification method to detect miRNA, which can simplify the operation process, improve the specificity and sensitivity of the test, and provide a more accurate and economical diagnostic method for clinical application.

In summary, the present investigation firstly explored miRNAs of parasitic origin from plasma samples of patients with EM infection by miRNA sequencing and used them for disease diagnosis, in addition, two new unlabelled micro RNA molecules were identified for the first time in the blood samples of patients with EM infection, respectively, named emu-miR-novel 1 and emu- miR-novel 2. And the clinical samples were validated by PCR with an AUC curve of 100%. The two novel micro RNA molecules are expected to be key molecules for the early diagnosis and therapeutic monitoring of multilocular echinococcosis.

## Supporting information

Ma et al. supplementary material Ma et al. supplementary material

## Data Availability

The data underlying the findings of this study are available upon request from the the corresponding author (X. A.) Due to privacy concerns related to the protection of research participants' identities, these data cannot be made publicly available.

## Supplementary material

The supplementary material for this article can be found at https://doi.org/10.1017/S0031182024001367
