# Supplementary figures and images for: Novel miRNA biomarkers for alveolar echinococcosis: sequencing and clinical validation
Source: Parasitology. 2024 Oct 18;151(13):1473–86. doi: 10.1017/S0031182024001367 (PMC12041903; doi:10.1017/S0031182024001367)

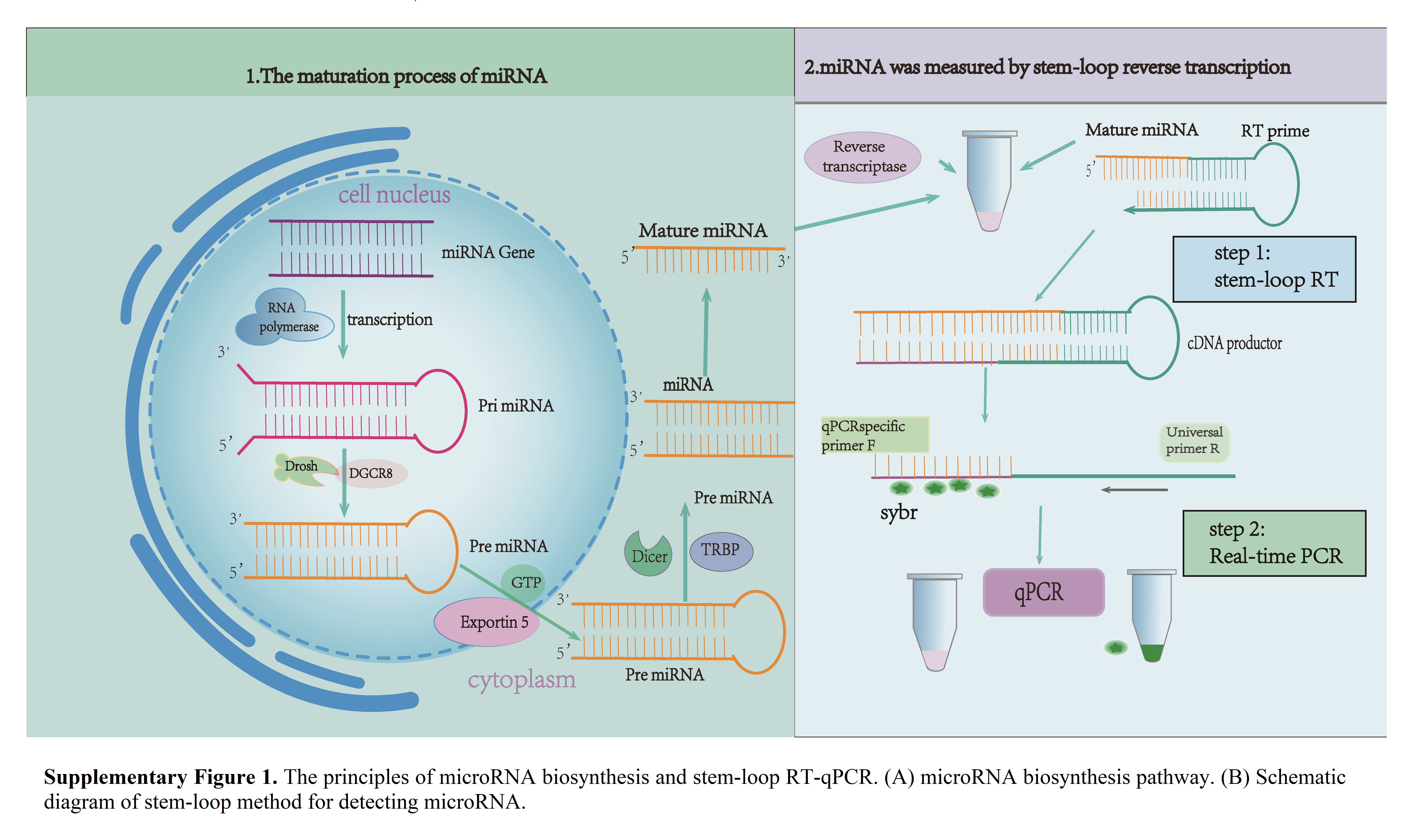

Supplement: Ma et al. supplementary material [file S0031182024001367sup001.tif]
